# Multifunctional thermoregulating and water repellent cellulosic textile†

**DOI:** 10.1039/d5gc00943j

**Published:** 2025-04-28

**Authors:** Zahra Madani, Hossein Baniasadi, Pedro E. S. Silva, Maija Vaara, Marike Langhans, Inge Schlapp-Hackl, Lars Evenäs, Michael Hummel, Jaana Vapaavuori

**Affiliations:** a Department of Chemistry and Materials Science, School of Chemical Engineering, Aalto University Kemistintie 1 Espoo Finland jaana.vapaavuori@aalto.fi; b Department of Chemical and Metallurgical Engineering, Polymer Synthesis Technology, School of Chemical Engineering, Aalto University Kemistintie 1 Espoo Finland; c Department of Bioproducts and Biosystems, School of Chemical Engineering, Aalto University Espoo Finland; d Department of Chemistry and Chemical Engineering, Chalmers University of Technology Gothenburg Sweden; e Wallenberg Wood Science Centre, Chalmers University of Technology Gothenburg Sweden; f FibRe – Centre for Lignocellulose-based Thermoplastics, Chalmers University of Technology Gothenburg Sweden

## Abstract

Enhancing thermal comfort in textiles can contribute to improved user well-being, both in wearable technology and everyday clothing. This study introduces thermoregulation properties by embedding a phase change material (PCM) into regenerated man-made cellulose fibers *via* the Ioncell® technology. Calorimetric analysis revealed that the incorporation of myristic acid as PCM enables the fibers to absorb and release thermal energy, providing dynamic thermal regulation in response to temperature changes. Specifically, the PCM-fiber containing 50% (w/w) myristic acid demonstrated a phase change melting enthalpy of 73 J g^−1^, with a melting temperature of 54 °C. The melting enthalpy remained largely stable even after 100 thermal cycling tests, highlighting the excellent durability of the PCM-incorporated textiles. Furthermore, the resulting thermoregulating textile was treated with a hydrophobic coating composed of octadecenyl succinic anhydride, resulting in an average water contact angle of 75°, after post-washing, demonstrating good water repellency. The developed fabric combines thermal regulation with water repellency through eco-friendly processes, offering a promising alternative to conventional functional textiles.

## Abstract 2

The figure shows the development of a multifunctional textile *via* dry-jet wet spinning of cellulose fibers with a phase change material, followed by octadecenyl succinic anhydride treatment to achieve hydrophobicity.

## Graphical abstract

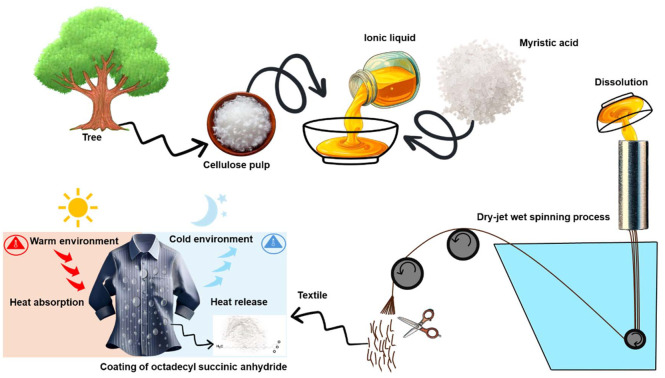

**Green foundation**

1. We showcase fully bio-sourced technical textiles, which are functionalized both by phase change and water repellent molecules, helping to regulate the temperature fluctuation and maintaining the thermal comfort of the user.

2. The material and fabrication method choices are guided by green chemistry principles. All the starting materials are renewable and non-toxic leading to a safer process. The spinning of the man-made cellulosic fibers employs ionic liquid as a less risky solvent than in conventional viscose or lyocell processes.

3. Further research could focus on validating the biodegradability and other end-of-life management strategies of the final textile fabrics, thereby reinforcing the principles of green chemistry throughout the product life cycle.

## 1. Introduction

Maintaining thermal comfort is essential for regulating metabolism and ensuring overall thermophysiological well-being.^1–3^ However, conventional everyday clothing often fails to adapt to temperature fluctuations throughout the day, leading to thermal discomfort from *e.g.* sweating or shivering. For instance, cotton clothing can hinder heat dissipation, aggravating heat stroke in hot weather, and hygroscopic fabric can become damp from sweat or rain, leading to freezing in cold weather.^4^ Therefore, the development of functional thermoregulatory textiles can support the user's thermal comfort.

A well-known approach involves integrating phase change materials (PCMs) into textiles.^5,6^ The technology for integrating PCMs into textiles was initially developed in the early 1980s by NASA with the aim of reducing the impact of extreme temperature fluctuations on astronaut space suits and gloves.^7^ PCMs such as paraffin wax,^8^ fatty acids (FAs),^9^ and fatty alcohols^10^ can absorb significant amounts of latent heat during solid–liquid phase transition and release the respective amount upon crystallization. Storing or releasing thermal energy helps to regulate temperature fluctuation.^10–12^

Previous studies on PCM-incorporated textiles have largely relied on fiber types derived from non-renewable resources or produced through processes that are neither environmentally friendly nor easily scalable. For instance, Nejman *et al.* developed a thermoregulating textile *via* coating of PCM microcapsules (MPCM) paste on polyester knitted fabrics. Based on their results, the phase transition enthalpy of 56–60 J g^−1^ was reported.^13^ Sarac *et al.* provided a cellulosic thermoregulating fabric including organic coconut oil encapsulated in melamine formaldehyde and polymethyl methacrylate shells, respectively. This enabled the fabrics to exhibit substantial latent heats ranging between 6.7 J g^−1^ and 14.9 J g^−1^.^14^ Baniasadi *et al.* introduced an electrospun nanofiber mat with thermoregulating functionality. The nanofibers were developed by electrospinning of polycaprolactone, including poly(ethylene glycol) as phase change particles. The fabricated mat possessed a latent heat of 61.7 J g^−1^ and reliable energy absorption-release cyclability over 100 heating–cooling cycles.^15^

Lu *et al.* fabricated a smart textile with a core–sheath structure based on paraffin wax as PCM in the core and polyacrylonitrile as a sheath. Moreover, hexagonal cesium tungsten bronze particle with excellent near-infrared region absorbing ability was utilized to enhance solar heating efficiency. The developed textile demonstrated a latent heat of 60.3 J g^−1^ after 500 heating–cooling cycles, illustrating its good stability.^16^ Qian *et al.* fabricated core–shell cellulose-based phase change fibers (PCFs) consisting of poly(hexadecyl acrylate) (PA16) as a PCM. PA16 was grafted to the cellulose fiber *via* UV irradiation, forming a core–shell-like structure. The resulting PCFs exhibited high phase change enthalpies of 73 J g^−1^, providing thermal-regulation properties.^17^

Building on existing literature, there is a need to advance the state-of-the-art by integrating PCMs with high thermal efficiency into fiber production processes that are scalable while maintaining sustainability. Leakage during phase transition is one of the main obstacles to implementing PCMs in everyday applications.^18^ Various solutions such as diverse spinning technologies,^19–21^ microencapsulation,^22,23^ and employing porous materials^24,25^ have been proposed to tackle the leakage issue.^26,27^

There are some reports of embedding PCMs into synthetic fiber and viscose-type fibers.^19^ Viscose fibers are characterized by high versatility resulting from the possibility of applying modification at various stages of the process,^28^ high elongation, and low production cost.^29^ However, the environmental impacts resulting from using petroleum-based resources as well as utilizing toxic chemicals in the process (*e.g.*, xanthation with carbon disulfide) pose limitations.^19^ The Ioncell® process is a Lyocell-type method for producing man-made cellulosic fibers (MMCFs) using ionic liquids (ILs) as cellulose solvents. Its milder conditions minimize cellulose degradation, resulting in higher fiber yield and improved strength, making it a sustainable alternative to existing MMCF production methods.^30^ Among various types of PCMs, bio-based fatty acids^31^ are particularly advantageous due to their high latent heat storage capacity and optimal phase transition temperatures.^32^ Additionally, FAs exhibit desirable properties such as chemical and thermal stability, non-toxicity, and cost-effectiveness, making them highly suitable for thermal energy storage applications.^33^

Water repellency in textiles, on the other hand, plays a crucial role in microenvironment regulation between the skin and external surroundings. Heavy sweating leaves humans feeling wet and sticky if the excessive fluids cannot be transported away through effective water repellency.^34^ There are several techniques for applying water-repellent coatings, including dip-coating, impregnation, padding, sol–gel, plasma, and spray coating.^35^

Cellulosic textiles have inherent hydrophilicity due to the abundance of hydroxyl groups, which can form hydrogen bonds with water molecules, allowing water to spread over the surface.^36^ Although fluorine-based or silane-based compounds have been used for water repellency in cellulose-based textiles, their toxicity and harmful environmental effects limit their applicability.^36^ For instance, serious health concerns have been observed for long-chain fluorocarbons resulting from bioaccumulation and biomagnification behavior.^36^ Resins such as polyurethanes, acrylates, silicon-based, and epoxy aqueous suspensions are commonly used in industrial applications to create superhydrophobic surfaces.^37^ For instance, studies have reported a water contact angle of 160° for alginate fabrics coated with hexadecyltrimethoxysilane and 152° for a polyester fabric treated with polydimethylsiloxane, a silicon-based coating.^35^ Liu *et al.* reported a water contact angle of 130°–135° by applying polyurethane to cellulose textiles.^38^ However, the environmental impact, durability concerns, and limited sustainability of common hydrophobic finishes have driven a growing demand for the development and adoption of eco-friendly hydrophobizing agents.^36,39^ Alkenyl succinic anhydrides (ASA), often with various chain lengths, are amphiphilic molecules that are water-resistant additives that meet the demands of hydrophobicity. The size of the alkenyl group and the degree of substitution are critical parameters in determining the level of hydrophobicity.^40^

Octadecenyl succinic anhydride (OSA) is the commonly used alkenyl succinic anhydride for the hydrophobization of hydrophilic materials and has been approved by the FDA for use in foods.^40^ In our previous work, we enhanced the compatibility of starch with polyamide by grafting OSA molecules onto the starch surface through esterification.^41^ Furthermore, Rivero *et al.* modified starch with OSA molecules *via* microwave-assisted reaction to develop low-density polyethylene/starch blends.^42^ The success of OSA-modified starch has renewed interest in the esterification of anhydrides with polysaccharides. OSA effectively modifies polysaccharides by reacting with their hydroxyl groups, as seen in cellulose, enabling functional enhancements through ester bond formation.^43^

This study introduces a novel approach to incorporating PCM into cellulose fibers *via* the Ioncell® process, achieving thermoregulation through a sustainable and scalable fiber-spinning method. To the best of our knowledge, there are no prior reports on PCM incorporation into the Ioncell® fibers for thermoregulatory functionality. By embedding PCMs like FAs into these fibers, we create thermoregulating textiles capable of storing and releasing heat energy in response to temperature fluctuations. These textiles not only demonstrate high latent heat but also maintain their functionality even under 100 heating–cooling cycles, offering significant long-term durability without the need for encapsulating the PCM. Moreover, we enhanced the fabric's water repellency by grafting OSA molecules onto the fabric, achieving a water contact angle of 75° after post-washing. The developed fabric combines thermal regulation with water repellency, making it a promising alternative to conventional functional textiles in the market.

## 2. Materials and methods

### 2.1 Materials

Birch prehydrolyzed kraft dissolving pulp (*η* = 446 mL g^−1^*M*_n_ = 50.3 kDa, *M*_w_ = 156.4 kDa, PDI = 3.1) provided by Stora Enso (Finland) as sheets was ground with a Wiley mill. The dry matter content of pulp was measured before dissolution in IL. The synthesis of IL was done by neutralizing 1,5-diazabicyclo[4.3.0]non-5-ene (99%, DBN, C_7_H_12_N_2_, CAS: 3001-72-7, *M* = 124.18 g mol^−1^, Fluorochem, UK) with an equimolar amount of acetic acid (100%, C_2_H_4_O_2_, CAS: 64-19-7, *M* = 60.06 g mol^−1^, Merck, Germany) under constant stirring at 70 °C as reported earlier.^44,45^ Myristic acid (C_14_H_28_O_2_) was obtained from Sigma Aldrich. Octadecenyl succinic anhydride (mixture of isomers) and 1,12-diaminododecane (≥98%) were purchased from TCI, Japan. Chloroform-d (99.8 atom % D) was obtained from Sigma-Aldrich.

### 2.2 Dope preparation

The cellulose dope was prepared based on the previous work.^46^ The molten IL was introduced to the preheated kneader at 80 °C. The dry cellulose concentration was adjusted to obtain 10%, and different amounts of MA, including 0%, 40%, and 50% (w/w) based on dry cellulose, were added to the IL, respectively. The concentrations of PCMs were selected to achieve the maximum possible PCM loading while maintaining feasible spinnability. The mixture was stirred at 30 rpm for 1.5 h under a vacuum of 60 mbar at 80 °C. To remove any undissolved cellulose and MA, the dopes were filtered through a layered filter mesh (GKD Ymax2, 6 μm nominal, Gebr. Kufferath AG, Germany, 6 μm) at 80 °C. The dope was stored at 5 °C before spinning.

### 2.3 Fiber spinning

The cellulose dopes, including various MA content, were spun *via* a customized dry-jet wet-spinning unit (Fourne Polymertechnik, Germany)^46^ into filaments by extrusion through a spinneret with a single hole of 0.1 mm in diameter and a length-to-diameter ratio of 0.2 at a temperature range of 65–68 °C. The filaments were passed through a 1 cm air gap and coagulated in a water bath at a temperature of 5 °C. Afterward, a Teflon roller guided the filaments to the godet, where the take-up velocity was adjusted to 11 m min^−1^ to obtain the draw ratio of 8.6. Subsequently, the fibers were washed in hot water (60 °C) three times and air-dried after washing.

### 2.4 Swatch preparation

The sample swatch was constructed with 9 vertical warp threads and 16 horizontal weft threads per centimeter (Fig. S1†). The warp threads were created by twisting a small bundle of fibers, following by twisting them into a two-ply thread. In contrast, the weft thread is an untwisted bundle of fibers to achieve a smooth and opaque surface. The swatch was handwoven using the bobbin lace technique, where each thread is attached to an individual bobbin, allowing independent movement. This technique requires two weft threads to work together, alternating over and under each warp thread to form a basic plain weave pattern.

### 2.5 Coating of cellulosic fabric with OSA molecules

To impart hydrophobicity to the fabric, we introduced a protective water-repellent layer by grafting OSA molecules into the fabric surface *via* a spray coating method. First, cellulose fabric was dried in the vacuum oven at 70 °C for 48 h. Then, a 1% OSA solution was prepared by dissolving OSA in chloroform and sprayed on the surface of the dried fabric. The fabric was then kept inside a preheated oven under a vacuum at 80 °C for 1 h.

### 2.6 Characterizations

The functional groups of MA present in the cellulose were analyzed *via* Fourier-transform infrared (FTIR) (PerkinElmer ATR FTIR instrument, USA), with wavenumber ranging from 4000 cm^−1^–500 cm^−1^, with a total average of 32 scans at a resolution of 4 cm^−1^. The morphology of the cross-section of spun fibers and the surface of the coated textile with OSA were examined by scanning electron microscopy (SEM) (Zeiss Sigma VP, Germany) at the acceleration voltage of 3 kV, sputtered with a 5 nm-thick layer of gold/palladium (Au/Pt).

The mechanical properties of spun fibers (*n* = 20) in both the dry state and wet state were assessed by Favigraph automatic single-fiber tester (Textechno H. Stein GmbH & CO., Germany) based on EN ISO 5079 standard (20 mm gauge length, pretension weight of 150 mg (DR 8.6), and 20 mm min^−1^ test speed, 20 cN load cell). All samples were conditioned overnight (20 ± 2 °C, 65 ± 2% relative humidity) before the measurements. The Young's modulus of the spun fibers was determined using the elastic region of the stress–strain curve following ASTM standard D2256/D2256Mf.

Thermogravimetric analysis (TGA) was conducted using a Netzsch STA 449 F3 Jupiter, heating the samples from 40 °C to 600 °C at a rate of 10 K min^−1^. The sample's phase change performance was assessed using differential scanning calorimetry (DSC) with a TA Instruments Discovery DSC 250 Auto. Approximately 5 mg of the sample was placed in an aluminum pan and subjected to heating. The final temperature was set to 100 °C, and the sample was then cooled to 0 °C. Both the heating and cooling cycles were carried out at a rate of 10 °C min^−1^ under a nitrogen flow rate of 40 ml min^−1^. The cycles were repeated twice, with the phase change enthalpies and melting point obtained during the second cycle. A cycling test involving 100 heating and cooling cycles was conducted for 10–50% to investigate the thermoregulating performance of the sample. The amount of loaded PCM was determined using the DSC results and calculated by eqn (1),^27^ where Δ*H*_m,pcm_ and Δ*H*_c,pcm_ represent the melting and crystallization enthalpies of the pure PCM (*e.g.*, MA), while Δ*H*_m,textile_ and Δ*H*_c,textile_ correspond to the melting and crystallization enthalpies of the PCM-fiber.1
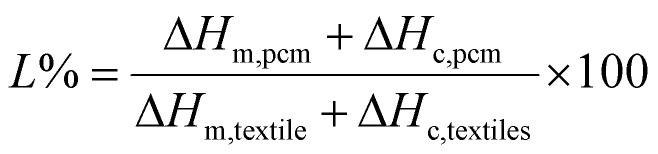


The leak test was conducted in an oven at 80 °C, which is relatively higher than the melting point of MA, for plain cellulose textile, pure MA, and all PCM-incorporated textiles for 4 h. The photographs before and after subjection to the oven were captured to visually evaluate any leakage of the PCM from textiles. Furthermore, to evaluate the phase change performance, thermal images were taken *via* a PIR uc 605 camera with a wide angle lens (focal length of 10 mm and a FOV of 59° × 46°), with an image resolution of 640 × 480 pixels and frame rate of 1 image per s. Focusing was performed manually using a ruler with distinctive markings due to differences in emissivity values. The heating source was a custom-built system composed of 28 quartz infrared heating elements, 1.5–8 μm wavelength, total power of 300 W, and a radiating area of 12.5 by 25 cm (Fig. S2†). A piece of fabric was set at a distance enough to ensure heating to 100 °C measured with the thermal camera. Afterwards, different samples were recorded while heated for 2 minutes and cooled for 3 minutes. Videos were generated to observe the experiments (Videos S1–S3†). The temperature measurements were done in a square region outside and inside the center of the samples. The grafted OSA% on the cellulose was quantified *via* elemental analysis (Thermo FlashSmart CHNSO elemental analyzer). Initially, the calibration curve was established based on the measured values of carbon (C), oxygen (O), and hydrogen (H) in the uncoated cellulose and the theoretical values for these elements in the anhydroglucose unit. The curve is illustrated in Fig. S3.† The measured elemental values in the OSA-*g*-textile were adjusted based on the calibration curve. Subsequently, the calibrated carbon value was used to measure the degree of substitution (DS) using eqn (2):2
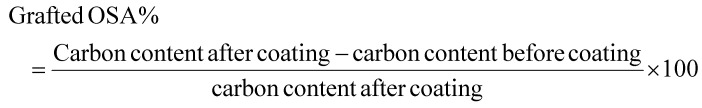


The surface wettability was assessed by a water contact angle test performed using a Theta Flex optical tensiometer (Biolin Scientific) and a 5 ml water drop to determine the water contact angle. The static contact angle after 1 second and 60 seconds was reported. The moisture regain (MR) was done on textile samples to assess the fiber's moisture absorption. In this regard, the samples were first dried to a constant weight in an oven at 105 °C for 4 h to remove all moisture. The dry weight of each sample was recorded (*W*_*d*_). The samples were then exposed to a controlled humidity environment (65–70% RH) for 24 h to allow for moisture absorption. After equilibration, the samples were weighed again to determine the wet weight (*W*_w_).3
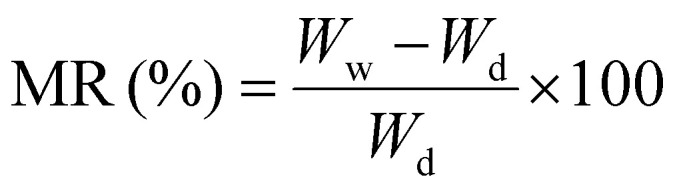


The durability of the OSA coating on the textile samples was assessed by performing a standard washing procedure. OSA-*g*-textile including 50% PCM was agitated in lukewarm water (40 °C) for 1 h. The sample was then air-dried at room temperature. After washing, the stability of PCM in the textile was assessed using DSC, while the durability of the OSA coating was examined through contact angle measurements.

## 3. Results and discussion

### 3.1 Chemical structure and mechanical properties

**Fig. 1:**
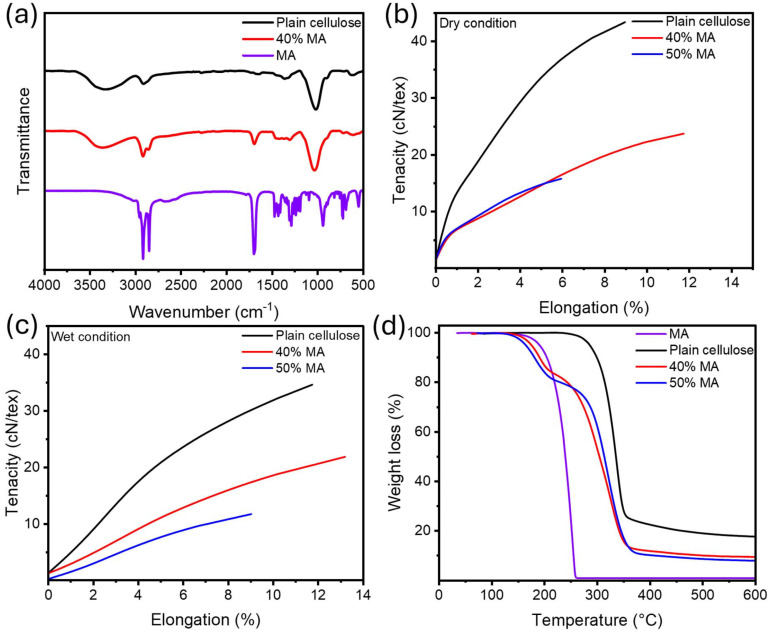
(a) FTIR spectra of MA, plain cellulose, and 40% MA. The mechanical properties of all samples in (b) dry condition and (c) wet condition. (d) TGA analysis of all samples.

The chemical structure was analyzed *via* FTIR spectroscopy, illustrated in Fig. 1a. Plain cellulose fiber showed transmittance peaks at 3339 cm^−1^ assigned to the hydroxyl group stretching.^47^ The transmission band at 2900 cm^−1^ corresponded to the stretching of the C–H group in the glucose unit. The peak at 893 cm^−1^ is characteristic of the β-glycosidic linkage between glucose units, while the signal at 1016 cm^−1^ is associated with the C–O stretching.^47^ In MA, the asymmetric and symmetric stretching vibrations of the C–H bond in the –CH_2_ group generate peaks at 2913 cm^−1^ and 2847 cm^−1^. The bending vibration of the C–H bond in the –CH_2_ group results in peaks at 1430 cm^−1^ and 1293 cm^−1^. The stretching vibration of C=O and the out-of-plane wagging vibration of –OH produce peaks at 1696 cm^−1^ and 936 cm^−1^, respectively. Additionally, the rocking vibration of –CH_2_, which occurs with four or more –CH_2_ groups in the chain, gives rise to the peak at 716 cm^−1^.^48^ It was observed that the characteristic absorption peaks of MA and plain cellulose appear across all MA-fiber (40% MA) spectra. The absence of new characteristic peaks in the MA-fiber spectra confirms that no chemical reactions occurred between MA and cellulose to form new chemical bonds.^49^

The mechanical properties are presented in Fig. 1b, c and Table 1 in both dry and wet conditions. Plain cellulose fiber exhibited a higher tenacity than other fibers due to the strong intra- and intermolecular hydrogen bonds between cellulose chains.^49^ The addition of MA into the cellulose caused a reduction in tenacity and Young's modulus of samples 40% MA and 50% MA, resulting from the disruption in the hydrogen bonding of cellulose chains.^27^ The reported tenacity of viscose fibers is 25 cN tex^−1^ in the dry state and 13 cN tex^−1^ in the wet state.^50^ In comparison, commercial lyocell fiber exhibits a higher tenacity of 36 cN tex^−1^ in the dry state and 29 cN tex^−1^ in the wet state.^50^ As indicated by Table 1, Ioncell fiber containing 40% MA falls within the tenacity range of viscose fiber, rendering it comparable to values reported in the literature.^19^

**Table 1:** Mechanical properties of all samples in both dry and wet conditions

Dry condition				Wet condition		
Sample	Tenacity (cN tex^−1^)	Elongation (%)	Young's modulus (MPa)	Tenacity (cN tex^−1^)	Elongation (%)	Young's modulus (MPa)
Plain cellulose	43.3 ± 1.9	9.0 ± 0.9	12.8 ± 0.1	34.7 ± 2.6	11.8 ± 1.1	5.3 ± 0.8
40% MA	23.7 ± 1.9	11.7 ± 1.0	5.2 ± 0.7	21.9 ± 1.6	13.2 ± 1.5	2.1 ± 0.6
50% MA	15.8 ± 1.6	5.9 ± 0.5	3.3 ± 2.5	11.8 ± 1.0	9.0 ± 0.8	1.9 ± 0.4

Moreover, the addition of MA up to 40% resulted in an increase in elongation at break due to the interruption of intermolecular forces between cellulose chains, leading to enhancement in chain mobility.^51^ This is also in line with the plasticization of cellulose films upon incorporation of MA, as reported previously.^51^ However, at higher concentrations of 50% MA, a significant reduction in elongation at break was observed, rendering fiber spinning unfeasible beyond this level. We speculate that at 50% MA, the additive reaches its saturation point, losing its plasticizing effect and consequently diminishing the elongation at break.

### 3.2 Fiber morphology

**Fig. 2:**
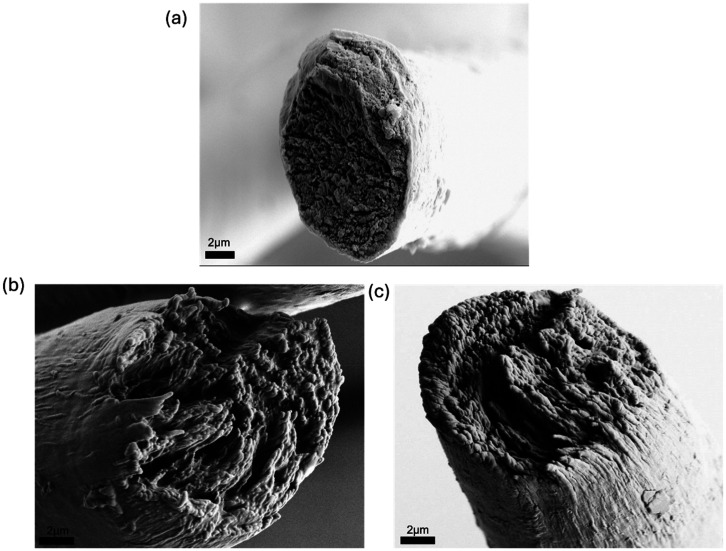
Cross-sectional SEM images (5kX) of (a) Plain cellulose, MA-fibers (b) 40% MA, and (c) 50% MA.

Fiber morphology is the key characteristic affecting the quality of the fabrics and apparel.^52^ The morphology of wet-spun fibers was analyzed using SEM to verify the uniform dispersion of MA within the cellulose matrix. Plain cellulose exhibited a fibrillar structure typical for Lyocell-type fibers (Fig. 2a). Upon 40% and 50% incorporation of MA, the fibrils get less pronounced showcasing even distribution of MA throughout the cellulose matrix without evidence of large agglomeration (Fig. 2b and c). This further supports the idea of MA intervening with hydrogen bonding between cellulose chains, resulting in plasticization and reduced tenacity, as discussed earlier.

### 3.3 Thermal analysis

TGA was carried out to characterize the thermal stability of all samples (Fig. 1d and Table 2). The plain MA and pristine cellulose fibers showed the maximum decomposition temperature of 250 °C and 337 °C, respectively, in alignment with the literature reported previously.^53^ Two distinct decomposition temperature ranges were observed for the fibers containing PCM, one of which is associated with the MA, and the other one corresponds to cellulose.^53^ The maximum decomposition temperature of MA dropped significantly in PCM-fibers compared to the plain MA. For instance, the decomposition temperature of MA in 50% MA sample was reduced to 188 °C as compared to 250 °C of the pristine MA. This can tentatively be explained by MA assuming different polymorph when crystallization upon wet-spinning process. Before, it has been reported that phase transition of γ-MA to the α-form reduces the melting temperature (*T*_m_), which in turn leads to the corresponding decrease in the decomposition temperature.^53^ Moreover, the addition of MA influences the maximum decomposition temperature of cellulose, causing it to shift to a lower temperature. As an example, plain cellulose exhibited a decomposition temperature of 337 °C; however, the incorporation of 50% MA led to the downward shift to 318 °C. This decrease in the decomposition temperature of cellulose may be attributed to the catalytic role of MA in promoting dehydration reactions or to the decreased supramolecular interactions between the cellulose chains.^54^ Importantly, the decomposition temperatures of the samples are significantly higher than the operating temperature range of the PCMs and the envisioned textiles, ensuring the thermal stability of the materials during phase change activation and everyday use.

**Table 2:** TGA results of all samples

Sample	Onset temperature^a^ (°C)	Onset temperature^b^ (°C)	Maximum decomposition temperature^a^ (°C)	Maximum decomposition temperature^b^ (°C)	Final mass residue (%)
MA	227	NA	250	NA	0
Plain cellulose	NA	307	NA	337	18
40% MA	169	272	187	320	10
50% MA	162	293	188	318	8

a MA

b Cellulose

**Fig. 3:**
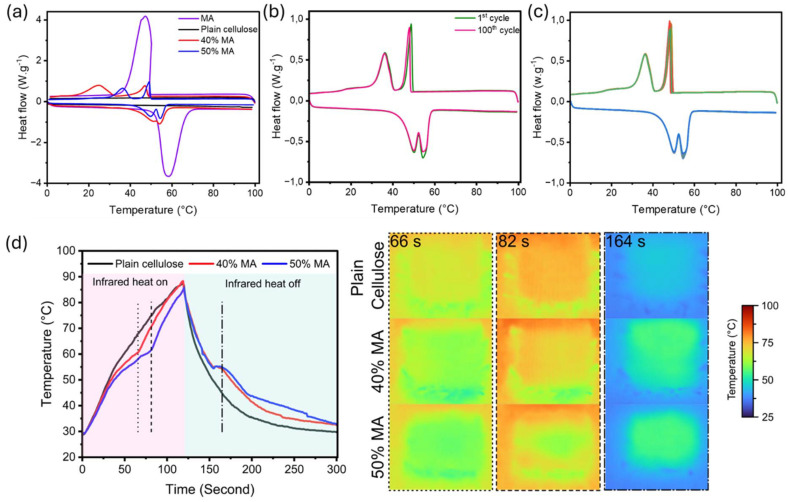
DSC analysis of (a) all samples, (b) the 1^st^ and 100^th^ cycles of 50% MA, and (c) the entire 100 cycles. (d) The temperature profiles of textile samples with different PCM compositions over a 300 s duration (heater turned off at 120 s). The thermal images snapshots from the samples were taken at different time frames of 66 s, 82 s, and 164 s.

PCMs are well known for their ability to absorb and release latent heat during phase transitions, making them effective thermal regulators to shield against fast temperature shifts. When the external temperature exceeds the PCM's melting point, heat energy is consumed in phase change, thus reducing heat transfer to the skin. Conversely, when the temperature drops below the crystallization point, the stored latent heat is released to moderate the cooling effect. As a result, PCMs can spontaneously absorb and release heat as needed to help stabilize the wearer's body temperature in varying conditions.^55^ These properties were investigated *via* DSC, as shown in Fig. 3a and Table 3.

**Table 3:** DSC analysis results of all samples

Sample	*T*_m_ a (°C)	Δ*H*_m_^a^ (J g^−1^)	*T*_m_ b (°C)	*T*_c_ a (°C)	Δ*H*_c_^a^ (J g^−1^)	*T*_c_ b (°C)	LR (%)
MA	58.5	194.3	NA	47.3	197.1	NA	NA
40% MA	54.1	52.9	51.4	47.1	13.7	24.6	23
50% MA	54.5	73.00	49.9	49.3	23.2	36.4	32
50% MA after leak test	54.4	72.4	49.7	48.8	23.1	36.2	NA
1^st^ cycle	54.6	67.9	50.4	48.9	22.6	36.3	NA
100^th^ cycle	54.9	64.4	50.3	36.4	19.8	36.2	NA

a Dominant peak

b Shoulder peak

Pristine MA demonstrated the endothermic peak at 58 °C and exothermic peak at 47 °C associated with solid–liquid and liquid–solid phase transitions in this temperature range, showing heat storage and heat release ability. As expected, neither endothermic nor exothermic peaks were observed for plain cellulose fiber within this temperature range. The addition of MA introduces two melting peaks and crystallization peaks due to the polymorphism of long-chain fatty acid crystals.^56^ The lower melting temperature shoulder peak likely represents a solid–solid transition, while the higher melting temperature peak corresponds to a solid–liquid transition.^57^

Incorporation of MA into cellulose resulted in a reduction of its melting temperature (*e.g.*, from 58 °C to 54 °C for 50% MA). This could be due to the weak intermolecular hydrogen bonding between MA and cellulose chains.^15^ The phase change enthalpies increased in proportion to the MA content, indicating that the heat storage capacity of the developed phase change fibers was dependent on the amount of PCM.

Melting enthalpy reduced from 194.3 J g^−1^ in the neat MA to 73.0 J g^−1^ in the sample containing the highest amount of MA, *i.e.*, 50% MA. The loading ratio of the MA was calculated *via*eqn (1) using the phase change enthalpies and is presented in Table 3. In both samples, the loading ratio was lower, although being more than 50%, than the nominal PCM loading, which could be due to restricted crystallization and MA loss during the washing process.^27^ The thermal cycling analysis was conducted on the PCM fibers, including the highest PCM content (*e.g.*, 50% MA), to ensure the thermal reliability of the sample over 100 thermal cycles (Fig. 3b and c). No significant changes in phase change temperatures and melting/solidifying enthalpies were observed after 100 cycles, demonstrating the excellent thermal storage/release ability of PCM fiber.

Our DSC analysis revealed that even at a relatively low effective PCM loading ratio, our fibers demonstrated excellent thermoregulating performance, surpassing or aligning with values reported in previous studies. For example, Konuklu *et al.* achieved melting enthalpies of 71.3 J g^−1^ and 32.7 J g^−1^ by incorporating MA into methylcellulose and hydroxyethyl cellulose composites, respectively.^58^ Similarly, Wu *et al.* developed electrospun nonwoven polyvinyl alcohol and PEG composites with a melting latent heat of 60.1 J g^−1^.^59^ Chen *et al.* reported a melting enthalpy of 70.8 J g^−1^ by embedding lauric acid into a polyethylene terephthalate composite. These comparisons highlight that the thermoregulatory performance of our fibers is not only competitive but also consistent with the benchmarks established in the literature.^60^ The distinct advantage of our chosen strategy is the capability to fabricate PCM-fibers by the Ioncell® process, which is both scalable and produces filaments that can be directly used in commodity textiles.

### 3.4 Leak test and thermal regulation performance of textile prototypes

To evaluate the thermal regulation properties of the developed fibers, several swatches were fabricated using PCM-fibers. Then, the thermal regulation performance of all swatches was assessed by subjecting them to heating and cooling cycles using an infrared heater. Fig. 3d presents the temperature profiles of textile samples with varying PCM compositions over a 300 s period (under an infrared heater on for 2 min/off exposure for 3 min). The graph shows the temperature changes at the midpoint of the samples during the heating phase and the cooling phase.

The addition of PCM significantly influenced the thermal performance of the textiles. Within 40 s, all samples rapidly reached 51 °C. As compared to the pure cellulose textiles, PCM-containing samples exhibited a delayed temperature increase, evidencing the thermal buffering effect.^27^ The delayed temperature rise in MA-textiles can be attributed to the latent heat absorption during the phase change from solid to liquid, effectively regulating the thermal energy transfer.^27^ During the cooling phase when the infrared lamp was turned off, the temperature of all samples dropped, however, a delay in cooling was detected from MA-textiles, indicating the latent heat release. In PCM-textiles, the observed plateau corresponds to the latent heat absorption and release process.^61^ These results confirm that the incorporation of MA enhances the thermal regulation performance of the textiles by mitigating rapid temperature fluctuations. This improvement is particularly significant for applications requiring stable thermal conditions, such as protective clothing, sportswear, or smart textiles for extreme environments.^17^

The leakproof test was done for all MA-textiles, as well as pure MA, to evaluate the strength of non-covalent bonding between MA and cellulose in the textile fibers. Samples were placed in an 80 °C oven to compare their leakage behavior. Digital photographs of the samples before and after the leak test are shown in Fig. S4.† As demonstrated, the pure MA completely melted, causing the blue paper underneath to become wet. In contrast, no signs of paper wetting were observed in the PCM textiles, indicating that the phase change process occurred within the fibers without significant leakage. The tested sample (50% MA) underwent DSC analysis to assess its phase change performance. The DSC graph is presented in Fig. S5,† with the corresponding data summarized in Table 3. No significant differences were observed in the phase change properties of the sample after the leak test, further confirming the absence of MA leakage during the phase change process.

### 3.5 Hydrophobization of thermoregulating textile and moisture regain

To further add functionality to the thermoregulating textiles, the percentage of OSA was measured *via* elemental analysis for the sample with better thermoregulation performance (*e.g.*, 50% MA), summarized in Table 4. The OSA grafted percentage was calculated as 1.8%, indicating approximately 1.8 OSA molecules are grafted per 100 anhydroglucose units. The grafted OSA made covalent bonds with the cellulose molecules, as confirmed by FTIR analysis (Fig. S6†). OSA grafted textile (OSA-*g*-textile) exhibited peaks resulting in the grafting of OSA molecules on cellulose through the covalent reaction between the cellulose hydroxyl group and the anhydride ring of OSA. Peaks at 2915 cm^−1^ and 2844 cm^−1^ resulted from grafted alkyl chain. Moreover, the peak at 1697 cm^−1^ is considered a characteristic peak of a carbonyl group in an ester bond (C=O), which confirms the esterification reaction. The C–C–O and O–C–C stretching bonds of ester groups at 1249 and 1024 cm^−1^ may overlap with the characteristic peaks of the anhydroglucose unit. The result here is aligned with the report presented in our previous work.^41^

**Table 4:** Elemental analysis results and the calculated amount of grafted OSA percentage on textile (OSA-*g*-textile). The data was calibrated by the equation shown in the Figure. S3†

Sample	Carbon (%)	Hydrogen (%)	Oxygen (%)	Grafted OSA (%)
50% MA(calibrated)	65.04	9.71	30.98	
OSA-*g*-textile (calibrated)	66.23	9.24	27.93	1.8

**Fig. 4:**
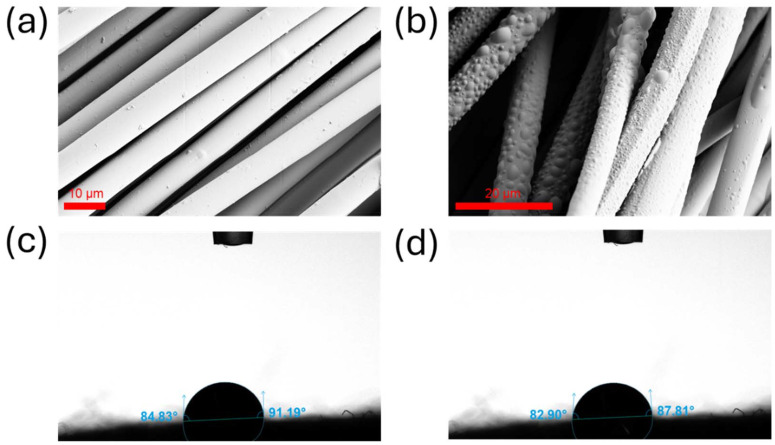
SEM analysis of textile (a) before and (b) after coating with OSA. Contact angle measurement of OSA-*g*-textile after (c) 1 s and (d) 60 s after the water droplet deposition.

The impact of surface treatment on the morphology of the photothermal textile was examined *via* SEM (Fig. 4a and b). The uncoated textile has a smooth surface. However, by the addition of OSA molecules, the round and oval geometry of OSA particles was attached to the surface of the fabric, providing a rough surface. It can be seen from Fig. 4b that OSA molecules were uniformly coated on the surface, indicating good interfacial adhesion between the cellulose surface and OSA particles. To investigate how grafted OSA particles enhanced the wetting properties of the cellulosic fabric, OSA-*g*-textile was analyzed through a water contact angle for a 60 s duration (Fig. 4c and d). The plain cellulose fiber readily absorbs water upon contact (with the droplet immediately absorbed, attributed to the abundance of hydroxyl groups in cellulose, Fig. S7†). In contrast, OSA-*g*-textile demonstrated an average water repellency of the fabric, which was reflected by a contact angle of 85° ± 1° after 60 s (Fig. 4d). OSA molecules include hydrophobic alkyl chains derived from the octenyl group, which reacts with hydroxyl groups on the cellulose, attaching hydrophobic chains to the cellulose surface, resulting in hydrophobicity of the surface against water absorption.^43^ Our result demonstrated that by only grafting 1.8% OSA particles to the surface of cellulose, we can obtain a good hydrophobicity in line with those reported in the literature. The conventional hydrophobic agents such as polyurethanes, acrylates, silicon-based and epoxy aqueous suspensions are commonly used in industrial applications to create superhydrophobic surfaces. However, they pose recyclability issues at the end of the textile's life.^35^ We believe that the grafted OSA content of 1.8% in our case does not significantly affect the recyclability of cellulose, while still imparting a moderate level of hydrophobicity to the cellulose textiles.

For instance, Yousafi *et al.* modified an all-cellulose nanocomposite (ACNC) with a silane coupling agent to enhance water repellency; however, this treatment compromised the cellulose purity in the composites. As a result, the highest contact angle achieved was 93°.^62^ Li *et al.* achieved a contact angle of 89° for plasticized starch/cellulose nanofibril (PS/CNF) nanocomposite films by incorporating 20% CNFs using the solution casting method.^63^ Balakrishnan *et al.* measured the water contact angle of 52° for thermoplastic starch/CNF (TPS/CNF) nanocomposite *via* loading of 3% CNF.^64^

The pure cellulose fabric exhibited the highest MR, around 9% ± 1%, due to the strong hydrophilic nature of cellulose, which readily absorbs moisture from the environment through hydrogen bonding with water molecules. In contrast, the incorporation of 50% MA significantly reduced the moisture regain to 6% ± 0.7%. This reduction is attributed to the hydrophobic nature of MA, which limits the accessibility of water molecules to the cellulose matrix. The most pronounced decrease in moisture regain was observed in the PCM fabric coated with OSA, which had an MR of only 1 ± 0.2%. The OSA coating introduces hydrophobic functional groups onto the fabric surface, effectively forming a moisture-resistant barrier that further inhibits water absorption. These results align with the contact angle measurements, which showed significantly higher surface hydrophobicity after coating the surface with OSA.

### 3.6 Washing durability and evaluation of PCM release

After washing the OSA-*g*-textile including 50% MA, the integrity of the hydrophobic coating was assessed with water contact angle measurement (Fig. S8†). It can be seen that the contact angle reduced to 75° after washing 1 h in warm water (40 °C). The DSC analysis of OSA-*g*-textile including 50% MA showed the melting enthalpy of 67.2 J g^−1^, showing no significant changes after post-washing (Fig. S9†).

## 4. Conclusions

This study presents a novel approach for developing thermoregulating textiles by integrating phase change material, directly into cellulose fibers using the Ioncell® process. The resulting PCM-fibers with the highest PCM content (nominally 50% MA) demonstrate good mechanical properties comparable with the viscose fibers as well as excellent thermoregulation properties, with phase change enthalpies reaching up to 73 J g^−1^ for the fibers containing 50% MA. These fibers effectively absorb and release thermal energy, enabling them to maintain temperature stability in response to environmental fluctuations. Notably, the fibers retained their thermoregulating performance even after 100 thermal cycles, highlighting their durability for long-term use. The integration of MA up to 50% concentration strikes a balance between flexibility and thermal regulation performance, making the fibers suitable for a wide range of applications, including smart textiles. The fibers also exhibited no leakage during thermal cycling, ensuring efficient heat storage and release. The applied hydrophobic coating achieved a water contact angle of 75° after post-washing, imparting the fabric with excellent water repellency. This is a significant advantage for outdoor applications, such as functional agrofabrics, where balancing thermal regulation with water resistance is critical. Overall, these results provide a new way of combining renewable non-toxic raw materials and a sustainable spinning process for producing thermoregulating water-repellent textiles for applications in extreme weather conditions. Future research could focus on optimizing the PCM loading to enhance thermal performance efficiency, exploring alternative sustainable PCMs, and assessing the scalability of this technology for industrial applications. Additionally, addressing the challenges of end-of-life management, particularly regarding the recyclability and biodegradability of PCM-containing functional fabrics, is essential to minimize the environmental impacts over the whole product life. Evaluating the long-term wearability, identifying the potential of using PCMs with lower phase change transition temperatures, and the comfort of these textiles under real environmental conditions will be essential for advancing their commercial viability.

## Data availability

The data supporting these findings are openly available at https://zenodo.org/records/15347991.

## Conflicts of interest

There are no conflicts to declare.

## Supplementary Material

GC-027-D5GC00943J-s001
