# Supplementary material for: Multifunctional thermoregulating and water repellent cellulosic textile
Source: Green Chem. 2025 Apr 28;27(22):6482–92. doi: 10.1039/d5gc00943j (PMC12082390; doi:10.1039/d5gc00943j)
Supplement: GC-027-D5GC00943J-s001 [file GC-027-D5GC00943J-s001.pdf]

Supplementary Information (SI) for Green Chemistry.
This journal is © The Royal Society of Chemistry 2025

Supplementary information

Multifunctional thermoregulating and water repellent cellulosic textile

Zahra Madani[1], Hossein Baniasadi[2], Pedro E. S. Silva[1], Maija Vaara[1], Marike Langhans[1], Inge Schlapp-Hackl[3], Lars Evenäs[4,5,6], Michael Hummel[3], Jaana Vapaavuori[1]*

[1]Department of Chemistry and Materials Science, School of Chemical Engineering, Aalto University, Kemistintie 1, Espoo, Finland

[2]Department of Chemical and Metallurgical Engineering, Polymer Synthesis Technology, School of Chemical Engineering, Aalto University, Kemistintie 1, Espoo, Finland

[3]Department of Bioproducts and Biosystems, School of Chemical Engineering, Aalto University, Espoo, Finland

[4]Department of Chemistry and Chemical Engineering, Chalmers University of Technology, Gothenburg, Sweden

[5]Wallenberg Wood Science Centre, Chalmers University of Technology, Gothenburg, Sweden

[6]FibRe – Centre for Lignocellulose-based Thermoplastics, Chalmers University of Technology, Gothenburg, Sweden

Corresponding author: jaana.vapaavuori@aalto.fi

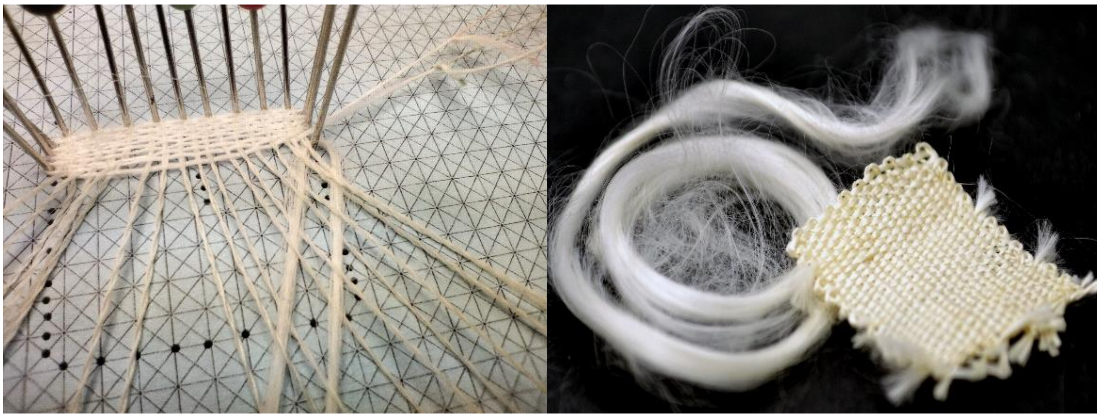

Figure S1. Preparation of swatch from developed fibers.

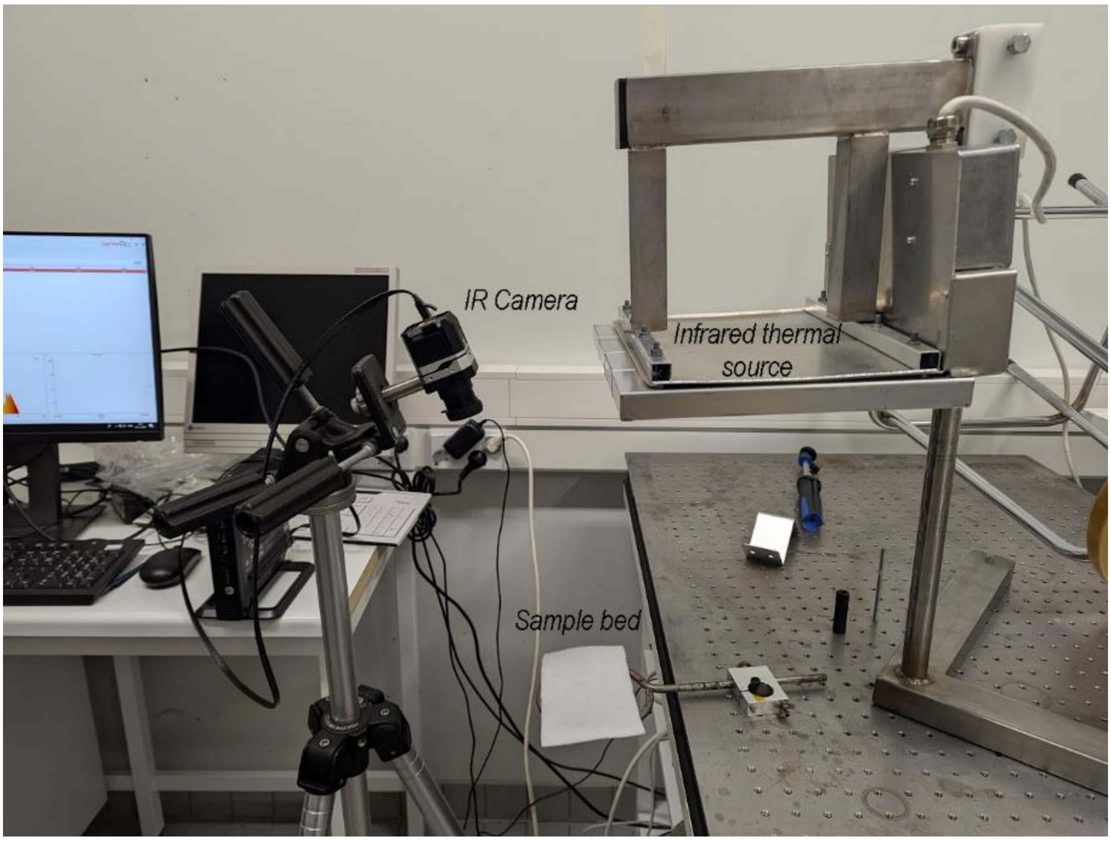

Figure S2. Thermal camera setup for thermal measurement.

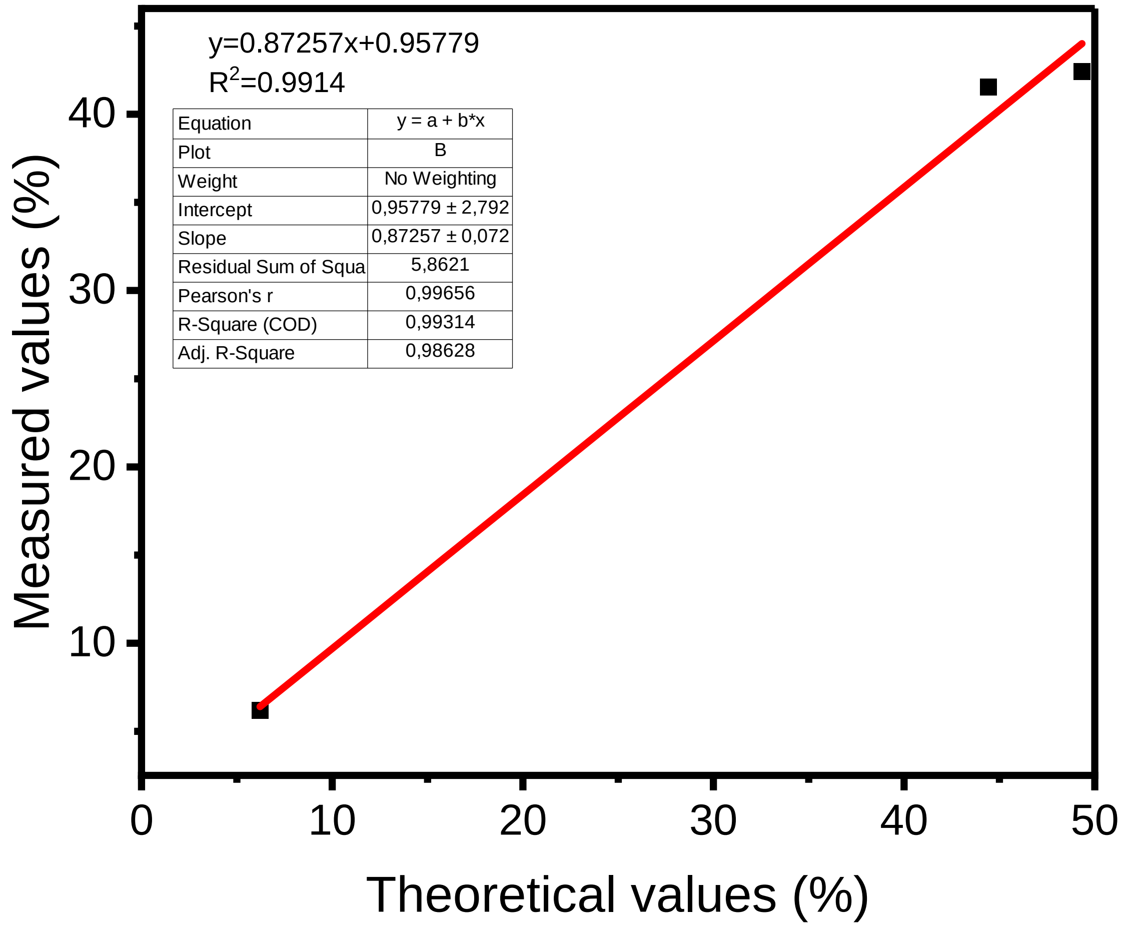

Figure S3. Cellulose elemental calibration curve.

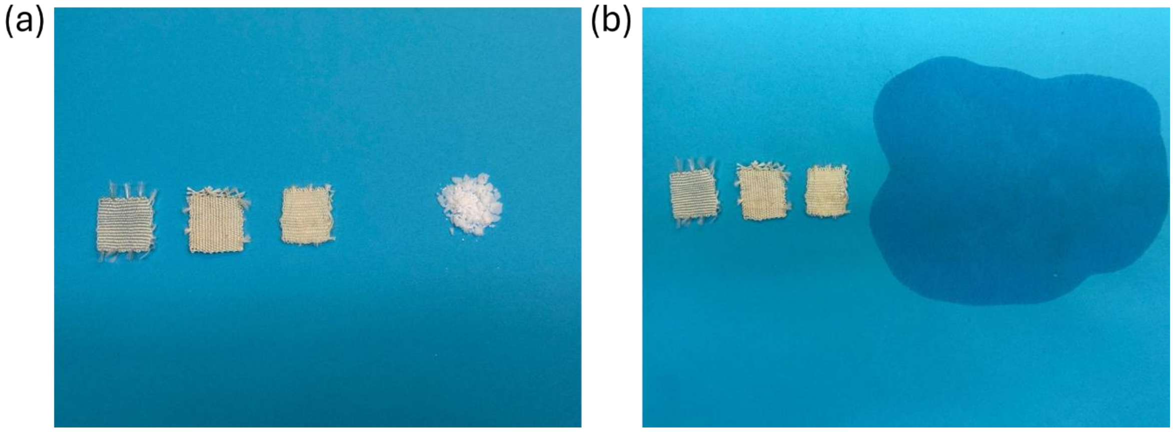

Figure S4. The leak test (a) before putting it in the oven, and (b) after putting it in the oven at 80°C for 4h.

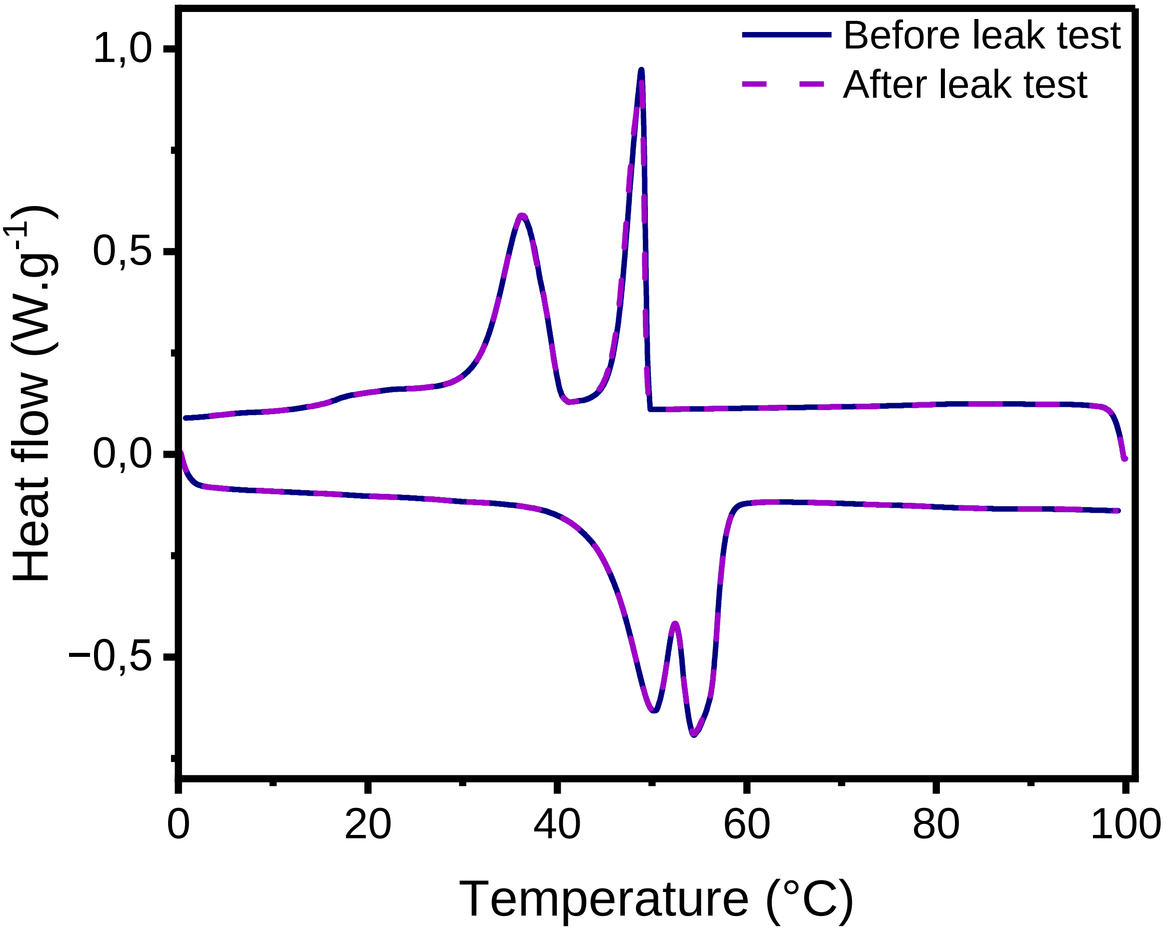

Figure S5. DSC analysis of the sample (50% MA) before and after the leak test.

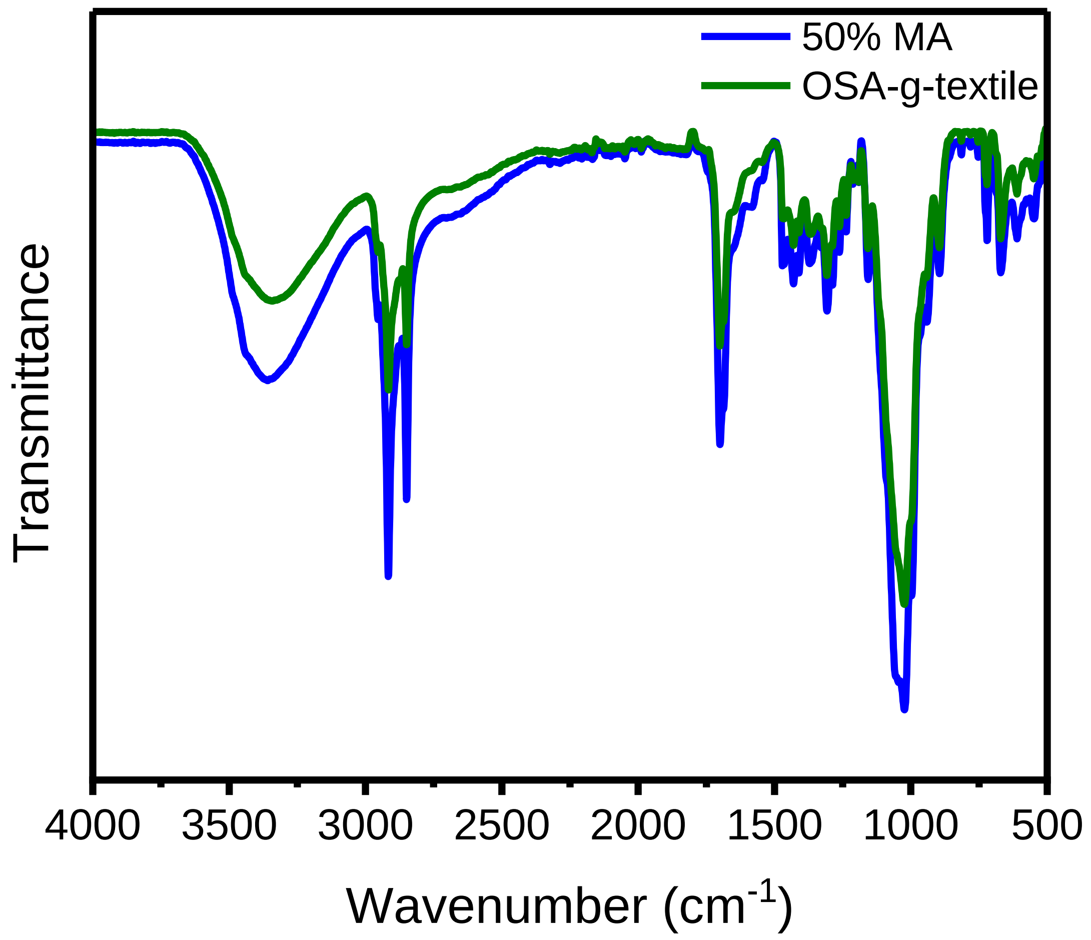

Figure S6. FTIR analys is of samples a) before coating, b)after coating.

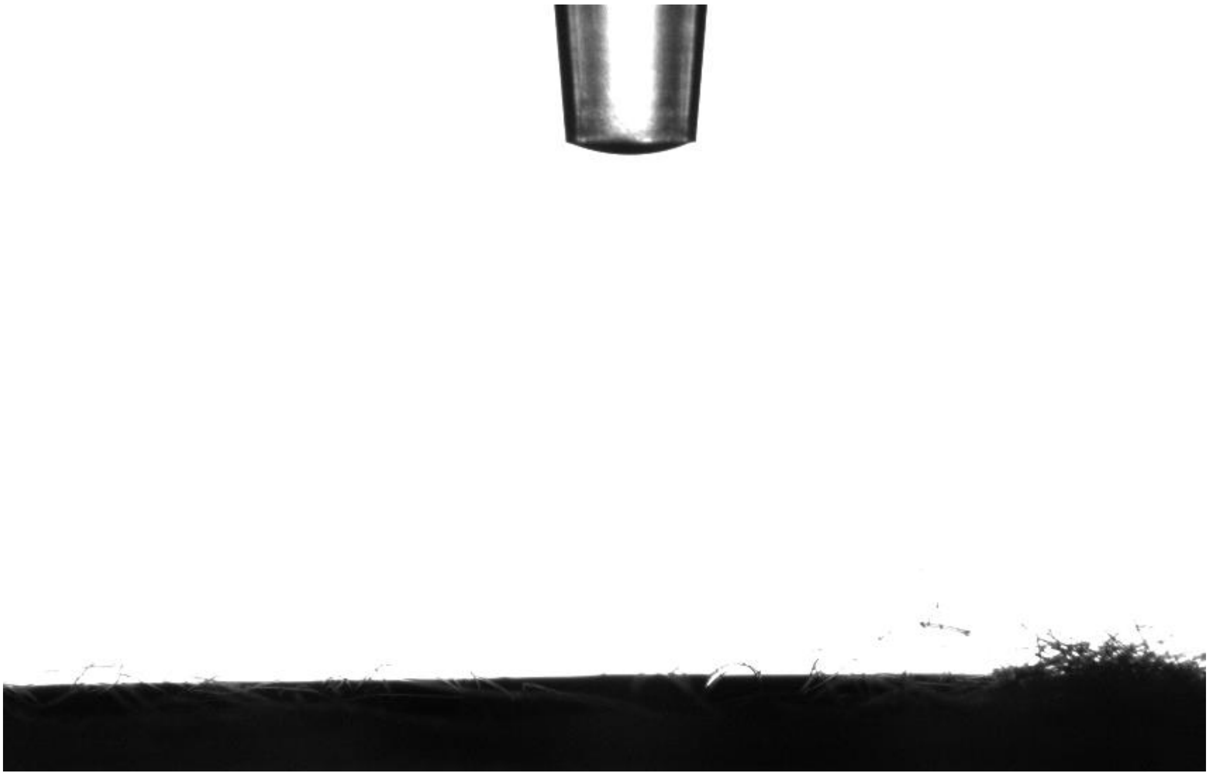

Figure S7. Water contact angle measurement of plain cellulose as soon as exposing to the water droplet.

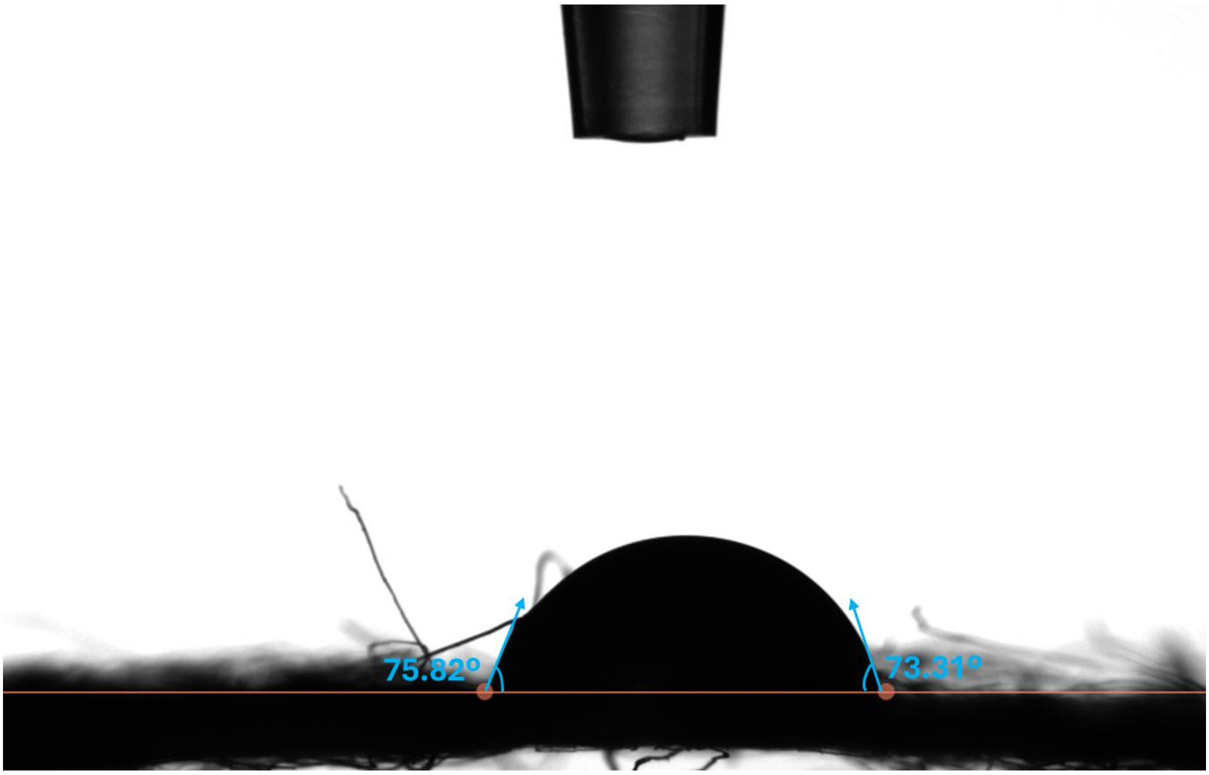

Figure S8. Water contact angle measurement of OSA-g-textile after washing test after 60 s.

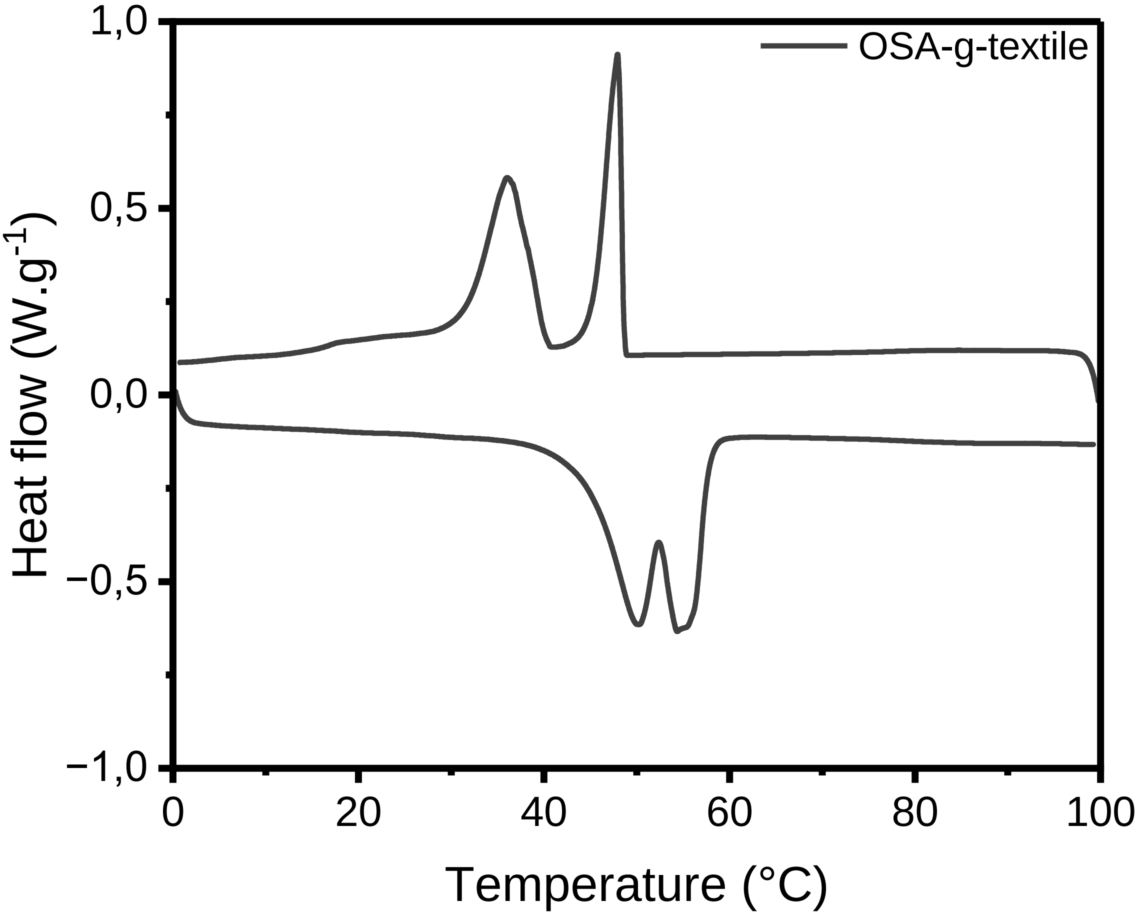

Figure S9. DSC analysis of OSA-g-textile including 50% MA after washing.
